# Mutation in the Cadherin Gene Is a Key Factor for Pink Bollworm Resistance to Bt Cotton in China

**DOI:** 10.3390/toxins14010023

**Published:** 2022-01-01

**Authors:** Ling Wang, Dong Xu, Yunxin Huang, Huazhong Zhou, Weiguo Liu, Shengbo Cong, Jintao Wang, Wenjing Li, Peng Wan

**Affiliations:** 1 Key Laboratory of Integrated Pest Management on Crops in Central China, Ministry of Agriculture and Rural Affairs, Hubei Key Laboratory of Crop Disease, Insect Pests and Weeds Control, Institute of Plant Protection and Soil Fertilizer, Hubei Academy of Agricultural Sciences, Wuhan 430064, China; wangl@hbaas.com (L.W.); xud@hbaas.com (D.X.); congsb@hbaas.com (S.C.); wjt217@126.com (J.W.); liwj@hbaas.com (W.L.); 2 School of Resource and Environmental Sciences, Hubei University, Wuhan 430062, China; y.huang@hubu.edu.cn; 3 General Station of Plant Protection, Hubei Province, Wuhan 430070, China; hbszbzz@163.com (H.Z.); lwg8705@163.com (W.L.)

**Keywords:** pink bollworm, Bt resistance, cadherin, F2 screening

## Abstract

Transgenic crops producing *Bacillus thuringiensis* (Bt) toxins are widely planted for insect control, but their efficacy may decrease as insects evolve resistance. Understanding the genetic basis of insect resistance is essential for developing an integrated strategy of resistance management. To understand the genetic basis of resistance in pink bollworm (*Pectinophora gossypiella*) to Bt cotton in the Yangtze River Valley of China, we conducted an F2 screening for alleles associated with resistance to the Bt (Cry1Ac) protein for the first time. A total of 145 valid single-paired lines were screened, among which seven lines were found to carry resistance alleles. All field parents in those seven lines carried recessive resistance alleles at the cadherin locus, including three known alleles, *r1*, *r13* and *r15*, and two novel alleles, *r19* and *r20*. The overall frequency of resistance alleles in 145 lines was 0.0241 (95% CI: 0.0106–0.0512). These results demonstrated that resistance was rare and that recessive mutation in the cadherin gene was the primary mechanism of pink bollworm resistance to Bt cotton in the Yangtze River Valley of China, which will provide a scientific basis for implementing targeted resistance management statics of pink bollworm in this region.

## 1. Introduction

Planting Bt (*Bacillus thuringiensis*) transgenic crops is one of the most effective ways to control agricultural pests worldwide [1]. Unlike chemical pesticides, Bt crops cause no harm to non-target organisms [2]. Thus, Bt crops serve as an important alternative to chemical pesticides that help protect biodiversity and promote the ecological regulation of farmland [3,4,5,6]. However, the main threat to the success of Bt crops is that the target pests may develop resistance to these crops, affecting the long-term efficacy of Bt crops in pest control [7,8,9,10]. Therefore, resistance management is an integral part of Bt crop-based pest control.

Understanding the genetic basis of pest resistance to Bt crops in the field is the first step toward developing an effective strategy of resistance management [10,11]. To ensure the timely adoption of a reasonable resistance management strategy, it is necessary to clarify the type and dominance of resistance alleles of pest populations to Bt crops in the field. The common methods of identifying resistance alleles include DNA screening, F1 screening and F2 screening [12,13]. The F1 screening involves selecting the F1 progeny developed from single-pair matings between field-collected individuals and a laboratory-selected strain with known resistance. In contrast, the F2 screening contains selecting the F2 offspring of two-parent families derived from wild-mated females or single-pair crosses between wild-collected males and females [14,15,16,17]. Both DNA screening and F1 and F2 screening methods can effectively identify rare resistance alleles [11,13,14,15,16,17]. However, DNA screening only explores the known mutation sites of resistance alleles and may miss resistance alleles from unknown mutation sites [13]. The F1 screening method requires a lab-selected strain with known resistance, and the type of resistance alleles selected by the F1 screening method is largely determined by the known resistant strain [18,19,20]. Compared to the F1 screening method, the F2 screening method has no such restrictions and is therefore useful to identify new types of resistance alleles in the field [11].

Transgenic cotton expressing Cry1Ac can effectively control pink bollworms in the United States, India and China, but with different resistance management strategies, the genetic basis of resistance evolution in pink bollworm populations has been shown to be different among these countries [10]. In India, pink bollworms not only evolved practical resistance to univalent transgenic cotton expressing Cry1Ac but also to bivalent transgenic cotton producing Cry1Ac and Cry2Ab, while field populations of pink bollworms in the United States and China did not develop practical resistance [3,10,21,22,23]. Although field populations of pink bollworms in the Yangtze River Valley of China are still susceptible to transgenic Bt cotton, several cadherin recessive alleles have been identified by DNA screening and F1 screening, demonstrating the potential for field-evolved resistance [3,13,18,19,20,24]. At present, mechanisms involving five types of resistance genes (cadherin, ATP-binding cassette gene, tetraspanin, MAPK4K4 gene and aminopeptidase-N gene) genetically linked with resistance to the Bt (Cry1Ac) protein in the field populations of six pest species have been deciphered [25]. However, all Cry1Ac-resistant individuals of pink bollworm derived from field populations in India or China by F1 screening, DNA screening or bioassays only harbored cadherin resistance alleles [13,18,19,20,22,24]. It is not clear whether there are other types of resistance alleles in field populations of pink bollworms. Hence, it is necessary to adopt F2 screening to provide comprehensive genetic resistance information from field populations.

In this paper, we conducted an F2 screening for alleles associated with resistance to the Bt (Cry1Ac) protein in pink bollworm (*Pectinophora gossypiella*) from China for the first time. We intend to clarify the type and dominance of resistance alleles, explore possible novel resistance alleles and estimate the frequency of resistance alleles in the field populations. The results are expected to increase the understanding of the genetic basis and provide a scientific basis for implementing targeted resistance management statics of field populations of pink bollworms in China.

## 2. Results

### 2.1. Resistance of Field Populations to Cry1Ac

The concentration of Cry1Ac (in µg per mL diet) that killed 50% of larvae (LC_50_) was 0.160 (95% CI: 0.125−0.189) for the population from Anqing, 0.180 (95% CI: 0.015−0.257) for the population from Qianjiang, 0.176 (95% CI: 0.028−0.245) for the population from Xinzhou and 0.077 (95% CI: 0.058−0.092) for QJ-S laboratory susceptible strain. The resistance ratios were 2.08, 2.34 and 2.29 for populations from Anqing, Qianjiang and Xinzhou, respectively (Table 1). Based on the conservative criterion of nonoverlap, the 2.08-fold resistance to Cry1Ac in the population from Anqing, relative to that of QJ-S, was statistically significant, while resistance to Cry1Ac in the populations from Qianjiang and Xinzhou was not statistically significant. No survival at the diagnostic concentration of Cry1Ac (10 μg/mL) was detected in the three collection sites or in the susceptible QJ-S strain.

### 2.2. Resistance Allele Frequency in Field Populations

A total of 193 single-paired lines with 66 lines for the population from Anqing, 71 lines for the population from Qianjiang and 56 lines for the population from Xinzhou were screened. Among those 193 lines, a total of 145 lines were valid (i.e., produced F2 offspring successfully), with 52 lines from Anqing, 54 lines from Qianjiang and 39 lines from Xinzhou (Table 2). Among the 145 valid single-paired lines, 7 lines carried resistance alleles, with 2 lines from Anqing, 3 lines from Qianjiang and 2 lines from Xinzhou (Table 2).

The observed survival of F2 progeny at a diagnostic concentration of Cry1Ac in the above seven single-paired lines ranged from 3.1% to 8.3% (Table 3). There was no significant difference between the observed survival and the expected survival (6.25%) for each of the seven single-paired lines (Fisher’s exact test, *p* = 0.25−0.62) (Table 3). This indicates that there was only one single recessive allele conferring resistance in each field-male parent of the above seven single-paired lines, yielding a total of seven resistance alleles. The frequency of resistance alleles was 0.0241 (95% CI: 0.0106–0.0512) across all three populations, 0.0192 (95% CI: 0.0033–0.0745) in the Anqing population, 0.0278 (95% CI: 0.0072–0.085) in the Qianjiang population and 0.0256 (95% CI: 0.0044–0.098) in the Xinzhou population (Table 2).

### 2.3. Identification of Resistance Allele Types of Field-Male Parents in F2 Surviving Single-Paired Lines

To further identify the type of resistance allele carried by the field-male parents of the above seven single-paired lines from the F2 screening, PCR detection was performed. The results show that the field-male parent AQ39 from Anqing and QJ68 from Qianjiang carried the resistance allele *r1*, AQ15 from Anqing and XZ53 from Xinzhou carried the resistance allele *r13*, QJ58 from Qianjiang carried the resistance allele *r15* and QJ43 from Qianjiang and XZ27 from Xinzhou did not carry *r1*, *r13*, *r14*, *r15* and *r16* (Table 4), indicating that both the field-male parents QJ43 and XZ27 carried a novel recessive cadherin allele or a recessive noncadherin resistance allele.

### 2.4. Recessive and Dominant Analysis of Cadherin Loci in the QJ43 and XZ27 Strains Selected from the F2 Screening

**Figure 1:**
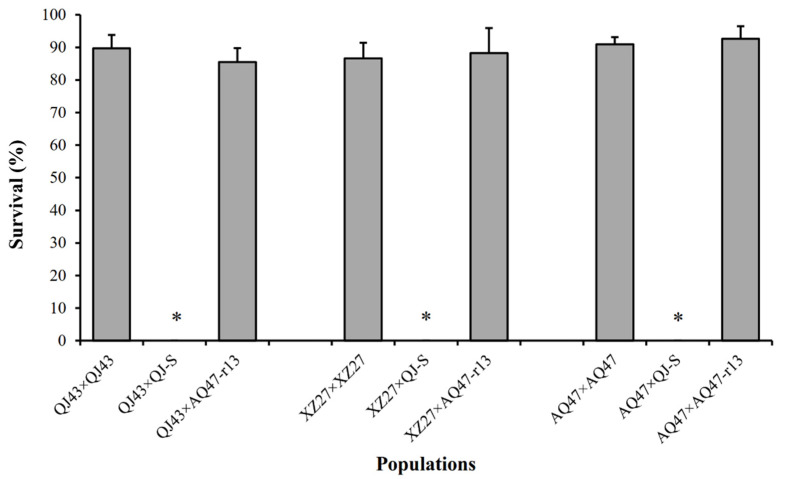
Survival at the diagnostic concentration of Cry1Ac of three resistant strains (QJ43, XZ27 and AQ47) and F1 progeny from crosses between each resistant strain and either the susceptible QJ-S strain or the resistant AQ47-r13 (the subset of AQ47 with the *r13r13* genotype) strain. Asterisks indicate 0% survival. Error bars show standard error.

Larval survival at a diagnostic concentration of Cry1Ac protoxins was 90% for QJ43 and 0% for QJ-S (susceptible larvae), The F1 progeny between QJ43 and QJ-S (Figure 1), which yielded a value of 0 for dominance parameter *h*, indicated completely recessive inheritance of resistance. At the diagnostic concentration, the survival of F1 progeny between QJ43 and AQ47 was 86% (Figure 1), which did not differ significantly from the 91% survival of AQ47-resistant homozygotes (Fisher’s exact test, *p* = 0.376), indicating that the QJ43 strain carried a recessive resistance allele, which we named *r19,* at the cadherin locus.

Larval survival at a diagnostic concentration of Cry1Ac protoxins was 87% for XZ27 and 0% for QJ-S (susceptible larvae). The F1 progeny between XZ27 and QJ-S (Figure 1), which also produced a value of 0 for dominance parameter *h*, demonstrated a completely recessive inheritance of resistance. At the diagnostic concentration, the AQ47-resistant homozygotes and F1 progeny between XZ27 and AQ47 did not differ significantly in larval survival (88% and 91%) (Fisher’s exact test, *p* = 0.645) (Figure 1), demonstrating that the XZ27 strain also carried a recessive resistance allele, which we named *r20,* at the cadherin locus.

### 2.5. Identification of Two Novel Cadherin Alleles (r19 and r20)

**Figure 2:**
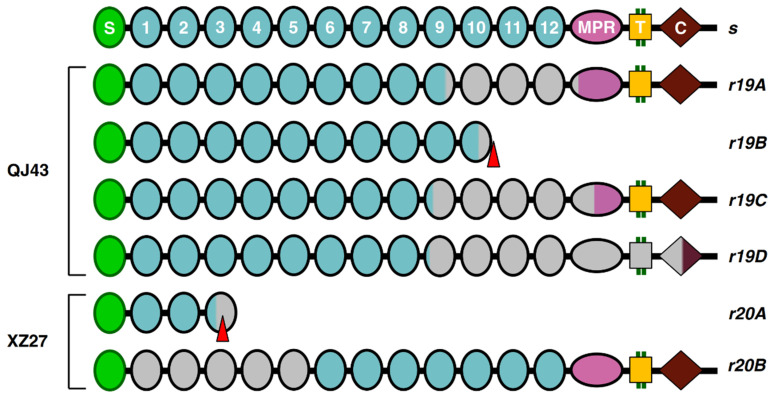
*PgCad1 r19* and *r20* mutation. The allele *r19* and *r20* and *s* allele protein alignment showing the amino–terminal membrane signal sequence (S), cadherin repeats (1–12), membrane-proximal region (MPR), transmembrane region (T) and cytoplasmic domain (C). Truncated structures indicate proteins predicted from cDNA with premature stop codons. Gray indicates missing regions of proteins caused by deletions.

Sequencing cDNA of *PgCad1* (the cadherin gene) from QJ43 revealed that *r19* has four different transcripts. Compared with the susceptible *s* allele of *PgCad1*, transcript *r19A* (GenBank Acc#MZ822470) has an 1158-bp deletion (3304–4461) encoding a PgCad1 protein that lacks 386 amino acids, 55 from cadherin repeat 9 (CR9), 318 from complete CR10-CR12 and 13 from the membrane-proximal region (MPR) (Table 5 and Figure 2). Transcript *r19B* (GenBank Acc# MZ822471) has an 1157-bp deletion (3629–4785) and a premature stop codon at base pairs 3835 to 3837, encoding a truncated PgCad1 protein missing 457 amino acids, including CR11-12, MPR, a transmembrane domain (TM) and cytoplasmic domain (CYT) (Table 5 and Figure 2). Transcript *r19C* (GenBank Acc# MZ822472) has a 1446-bp deletion (3117–4562) encoding a PgCad1 protein that lacks 482 amino acids, 118 from cadherin repeat 9 (CR9), 318 from complete CR10-CR12 and 46 from the membrane-proximal region (MPR) (Table 5 and Figure 2). Transcript *r19D* (GenBank Acc# MZ822473) has a 1956-bp deletion (3074–5029) encoding a PgCad1 protein that lacks 652 amino acids, 132 from cadherin repeat 9 (CR9), 448 from complete CR10-TM and 72 from CYT (Table 5 and Figure 2).

Sequencing cDNA of *PgCad1* from XZ27 demonstrated that *r20* has two different transcripts. The full-length transcript *r20A* (GenBank Acc# MZ822474) is 4421 bp, with an 881-bp deletion (1010–1890) and a 94-bp insertion in the deletion site, which encodes a truncated PgCad1 protein missing 1375 amino acids, including a partial CR3 and complete CR4-CYT (Table 5 and Figure 2). Transcript *r20B* (GenBank Acc# MZ822475) has 3717 bp in full-length cDNA with a 1585-bp deletion (306–1890) and a 94-bp insertion in the deletion site encoding a PgCad1 protein missing 497 amino acids, including CR1-5 (Table 5 and Figure 2).

**Figure 3:**
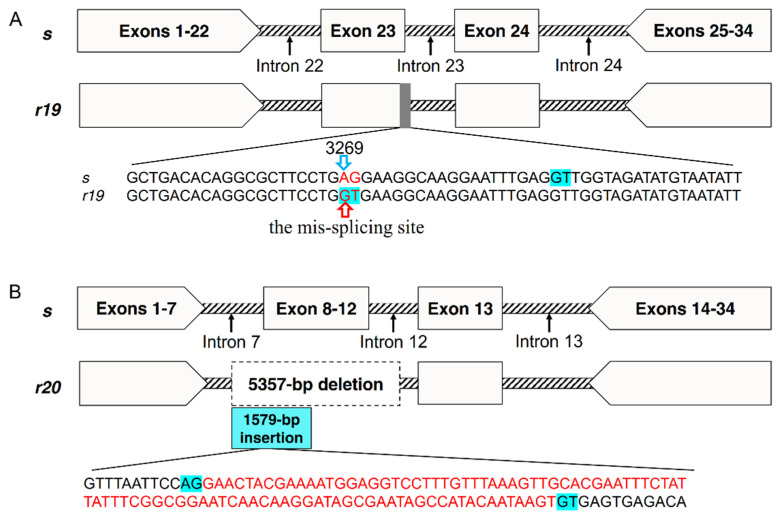
Map of the *PgCad1 r19* and *r20* mutation. (**A**) *r19* allele of *PgCad1*. The red sequence AG/GT indicates the mutation site in exon23. The blue background GT indicates splicing sites. The upward red arrow indicates the mis-splicing site in *r19*. The 3269th base represents the starting site of the common deletion sequence of the four transcripts of *r19*. (**B**) *r20* allele of *PgCad1*. The dotted box indicates a 5357-bp deletion from intron 7 to intron 12 in *r20*. The blue box indicates a 1579-bp insertion in the deletion site of *r20.* The blue background GT/AG indicates splicing sites. The red sequences indicates the 94-bp insertion in the two transcripts of *r20*.

Sequencing partial genomic DNA (gDNA) of *PgCad1* from QJ43 revealed that the *r19* allele has two base substitutions in exon 23. The bases AG (3269 to 3270 in cDNA of *s*) are mutated into GT (Figure 3A), which produces a mis-splicing site and eventually leads to sequence deletion of the transcript in *r19*. Compared with the *s* allele, there are no mutation sites in exon 22 and exon 24-29 of *r19*. However, introns of *r19* have obvious variation relative to *s*, especially if a 3431-bp fragment is inserted into intron 24 of *r19* (Figure S1). Sequencing partial gDNA of *PgCad1* from XZ27 demonstrated that *r20* has a 5357-bp deletion from intron 7 to intron 12, including partial intron 7, complete exon 8 to exon 12 and partial intron 12 (Figure 3B and Figure S2). In addition, a 1579-bp fragment is introduced at the deletion site of *r20*, which produces an additional exon with 94-bp in the two transcripts of *r20* (Figure 3B and Figure S2).

## 3. Discussion

Here, we present the results of an F2 screening for identifying Bt resistance alleles in pink bollworms collected from the Yangtze River Valley of China in 2017. The F2 screening method could theoretically identify any resistance allele. However, each of the seven individuals (field parent) identified by the F2 screening carried only one recessive resistance allele at the cadherin locus in 2017. Combined with previous studies, all resistant individuals identified by F1 screening and DNA screening carried recessive cadherin alleles in 2012–2015 [13,18,19,20,24], demonstrating that the gene mutation of pink bollworm in the field population mainly occurred in the cadherin gene locus and that recessive mutation in the cadherin gene is the primary mechanism of pink bollworm resistance to Bt in this region. Similarly, Cry1Ac-resistant individuals of pink bollworm screened from the field by bioassays in India also mainly carried cadherin mutant alleles [22]. It seems the cadherin gene mutation is probably the least costly in exchange for pink bollworm resistance to Bt (Cry1Ac) under field conditions.

The cadherin alleles identified by the F2 screening included three known resistance alleles (*r1*, *r13* and *r15*) and two novel resistance alleles (*r19* and *r20*), which were both different from the known resistance alleles *r1*–*r18* [18,19,20,22,24,26,27]. The novel resistance alleles *r19* and *r20* produced four and two transcripts, respectively, which implicated alternative splicing of mRNA, which was similar to the resistance alleles *r5*, *r7*–*r12* and *r15* [20,22]. Five transcripts, *r19A*, *r19B*, *r19C*, *r19D* and *r20A,* produced a mutant PgCad1 protein missing CR11-12 or more than CR11-12. The CR11-12 domain was the Cry1Ac-binding domain [28], which indicated that the resistance mediated by the above five transcripts was probably due to the decreased binding ability of the mutant PgCad1 protein to Cry1Ac. The transcript *r20B* produced a mutant PgCad1 protein without CR1-5 domains that cannot perform its normal task because the partial deletion of the CR5 domain of PgCad1 is associated with Cry1Ac resistance by decreased translation, increased degradation or mislocalization [24]. In addition, the gDNA mutation sites of the novel resistance alleles *r19* and *r20* were also different from the known resistance alleles *r1*–*r18* [18,19,20,22,24,26,27]. For *r19*, the starting point of the common deletion sequence of the four transcripts (3269–4461, partial exon 23 to complete exon 29) was undoubtedly caused by two base substitutions in exon 23, which produced a mis-splicing site. However, the sequences of exons 24, 25, 26, 27, 28 and 29 in the gDNA of *r19* did not change; therefore, the deletion mechanism of exons 24–29 in corresponding transcripts was not clear. Intron variations probably contribute to the resistance of *r19*. For *r20*, the 5357-bp deletion from intron 7 to intron 12 in gDNA led to the deletion of exons 8–12 in corresponding transcripts, which was similar to that of alleles *r1*, *r2*, *r9*, *r12* and *r13* [18,22,26]. The difference was that a 1579-bp sequence was inserted into *r20*, resulting in the introduction of an additional exon into corresponding transcripts. 

The results of the bioassay here suggested that the field populations of pink bollworms in the Yangtze River Valley of China still maintained good susceptibility to Bt cotton in 2017. Although bioassays can detect individuals with resistance caused by any mechanism, heterozygotes carrying recessive resistance alleles cannot be detected because the response of heterozygotes to Bt is similar to that of susceptible individuals [18,19,20,24]. No survivors at a diagnostic concentration of Cry1Ac were detected in the field in 2017, indicating that there were neither individuals with a dominant resistance allele nor homozygous individuals with a recessive resistance allele. Only heterozygous individuals with a recessive resistance allele or susceptible individuals did not differ from the bioassay results in 2012 to 2015. No survivors (a total of 2592 larvae tested) were detected at this concentration [3]. This finding was also consistent with the results of the F2 screening, in which all individuals carrying resistance alleles were recessive heterozygotes.

The results here confirmed that resistance was rare because the frequency of resistance alleles based on the F2 screening was 0.0241 (95% CI: 0.0106–0.0512) in China in 2017. Although the total frequency of seven *r* alleles (*r1*, *r2*, *r3*, *r13*, *r14*, *r15* and *r16*) was 0.0049 (95% CI: 0.0042–0.0057) from 2012 to 2015 based on DNA screening [13], this does not mean that the frequency of resistance alleles increased in 2017 compared with that from 2012–2015. DNA screening can only identify individuals carrying known resistance alleles and thus may underestimate the frequency of resistance, while F2 screening can detect any resistance allele, including individuals carrying unknown resistance alleles [13,29]. In fact, two novel resistance alleles were found in the F2 screening, further suggesting that DNA screening probably underestimates the frequency of resistance alleles. Therefore, F2 screening results better reflected the actual frequency of resistance alleles in the field than DNA screening results.

In the Yangtze River Valley of China, pink bollworm resistance to Cry1Ac increased from 2005–2007 and 2008–2010 due to the large area planted in Bt cotton and lack of refuges. In contrast, the figures decreased from 2008–2010 and 2011–2015 because of the large area planted in second-generation Bt cotton, which provided enough refuges [3,30]. The results of the F2 screening confirmed that the resistance allele frequency of pink bollworms in the field population was mainly attributed to a small number of recessive heterozygous individuals, which explains why the refuge strategy can effectively delay the development of resistance of pink bollworms to Bt in this region. Different resistance management strategies were performed in the United States, India and China, which led to different consequences [3,10,22,31], indicating that a reasonable resistance management strategy is essential to delay the resistance of pink bollworms to Bt. It is vital to understand the genetic basis of pest resistance to Bt crops in the field to formulate a reasonable resistance management strategy. Although the F2 screening showed that mutation in the cadherin gene was the main adaptative mechanism of pink bollworm to Bt cotton in the Yangtze River Valley of China, other resistance genes such as ATP binding cassette transporter genes [32] or resistance mechanisms such as changes in immune systems [32] could not be completely excluded. Therefore, we must strengthen resistance monitoring and rapidly gain a better understanding of the dynamics of resistance genes in the field.

## 4. Materials and Methods

### 4.1. Insects and Bt Proteins

We used eight laboratory strains of pink bollworm: a susceptible strain (QJ-S) and seven Cry1Ac-resistant strains (AZP-R, AQ47, AQ189, JL46, AQ65, QJ43 and XZ27). The QJ-S strain originated from the Yangtze River Valley, China and was reared in the laboratory without exposure to insecticides or Bt proteins. The AZP-R strain originated from Arizona with the cadherin genotype *r1r1*, while the AQ47, AQ189, JL46 and AQ65 strains were selected from the Yangtze River Valley of China with cadherin genotypes *r13r13*, *r14r14*, *r15r15* and *r16r16*, respectively [18,19,20,24,26]. The QJ43 strain was derived from a single-pair cross between a field-collected male (#43) from Qianjiang and a susceptible female from QJ-S, while the XZ27 strain was created by a single-pair cross between a field-collected male (#27) from Xinzhou and a susceptible female from QJ-S. The F1 progeny of those two crosses were reared on a diet without Bt protein, and the F2 offspring were selected on a diet with 10 μg/mL Cry1Ac. Survivors were collected to establish a new strain.

Third-generation larvae were collected from raw cotton purchasing stations in Anqing in Anhui Province as well as Qianjiang and Xinzhou in the Hubei Province of the Yangtze River Valley of China in October 2017. The collected larvae were placed in an incubator at 28 ± 1 °C and a photoperiod of 14:10 (L:D) to break diapause. After pupation, males and females were distinguished under a microscope to prepare for the follow-up experiment.

Larvae and adults were maintained at 28 ± 1 °C and a photoperiod of 14:10 (L:D). The relative humidity (RH) was 50 ± 10% for larvae and 70 ± 10% for adults. Larvae were reared on a wheat germ artificial diet. To maintain resistance, approximately 600–720 larvae of AQ47 were fed a diet with 10 μg Cry1Ac per mL every fifth generation. For all dietary exposure of larvae to Bt toxins, F2 screening and general selection, we applied the protoxin form of Cry1Ac, which was purchased from Zhongbao Biotechnology Company, Beijing, China. Cry1Ac protoxin was expressed in the Bt HD73 strain, then purified, and its concentration was measured by the bovine serum albumin (BSA) determination method.

### 4.2. Bioassays

Approximately 100 newly emerged virgin adults (males and females in total) from each collection point described above were transferred to a cylindrical glass container (10 cm in diameter × 12 cm in height) to produce progeny. A piece of white art paper purchased from Wenzhou Snow Mountain Paper Co., Wenzhou City, Zhejiang Province, China, was placed on top of each glass container to collect eggs. Eight percent honey water was provided in each container and was changed every day.

Larval susceptibility to Cry1Ac was evaluated using diet incorporation bioassays. The stock dilution was added to liquid wheat germ diet in the amounts necessary to create final concentrations of 0 (control), 0.05, 0.1, 0.15, 0.2, 0.3, 1 and 10 μg Cry1Ac per ml solution for QJ-S, and 0, 0.05, 0.1, 0.2, 0.4, 0.8 and 10 μg Cry1Ac per ml for populations from each collection point. The diet was made in 200 milliliter batches of each concentration, cooled, shredded into pieces (ca. 1 by 1 by 1 cm) and dispensed into 24-well culture plates with one piece of diet per well. The newly hatched larvae were placed individually in each well. For each population, each concentration was repeated three times, with 24 larvae per repeat (one culture plate). Plates were placed in an artificial climate chamber at 28 ± 1 °C with a photoperiod of 14:10 (L:D) h. After 21 days, all live fourth instars and pupae were scored as survivors.

### 4.3. F2 Screening

The male individuals collected in the field and the female individuals of the susceptible strain (QJ-S) were paired one by one. One pair of newly emerged virgin adults was transferred to a plastic cup (4 cm in diameter × 6 cm in height) that contained two 200-μL centrifuge tube caps. A piece of white art paper described above was placed on top of each cup to collect eggs. The caps in each cup were filled daily with 8% honey water as a nutritional supplement for the adults. The newly hatched larvae of F1 offspring of each single pair were reared on a conventional artificial diet until pupation. The newly emerged virgin adults of each F1 offspring were transferred to a cylindrical glass container (10 cm in diameter × 12 cm in height) to produce their own F2 progeny. For each F2 family, approximately 72 to 144 larvae were tested on a diet with the diagnostic concentration of Cry1Ac (10 μg/mL) using the bioassay method described above. Survivors for each F2 family were collected to generate F3 progeny and establish strains.

The expected survival of F2 offspring on a diet with Cry1Ac depends on the genotypes of the field-male parent. If the male parent has no resistance alleles, the expected survival of F2 offspring is 0%. If the male parent had one single recessive allele conferring resistance, the survival of F2 offspring is theoretically 6.25% (1/16) [11,33]. If survival was significantly greater than 6.25%, it was implied that the field-male parent had two recessive resistance alleles or one single non-recessive allele.

### 4.4. Detection of r Cadherin Alleles for Field-Male Parents of Resistant Strains Isolated from the F2 Screening

For all resistant strains isolated from the F2 screening, DNA molecular detection was conducted to identify whether their field-male parents carried *r* (*r1*, *r13*, *r14*, *r15* and *r16*) cadherin alleles. Individuals from the five Cry1Ac-resistant strains of pink bollworms described above were applied as sources of cadherin alleles for positive controls in PCR. The allele-specific primers for CK (a control for amplifiable *PgCad1* DNA), *r1*, *r13*, *r14*, *r15* and *r16* were the same as previously described [18,19,20,24,34]. DNA extraction of all samples was carried out according to the instructions of the Universal Genomic DNA Kit (CW2298M, CWBIO). PCRs were performed on a thermocycler using 2×Es PCR Taq (CWBIO, Beijing, China) in a 20 μL system containing 10 μL Es Taq, 0.4 μM forward primer and reverse primer, 50–100 ng gDNA and sufficient ddH_2_O. The PCR conditions for each *r* allele were the same as previously described [13,18,19,20,24,34].

### 4.5. Dominance of the Cadherin Locus for Resistant Strains Isolated from the F2 Screening

We crossed each resistant strain isolated from the F2 screening without known alleles (*r1*, *r13*, *r14*, *r15* and *r16*) with the susceptible QJ-S strain and separately with the resistant AQ47 (*r13r13*) strain. Seventy-two F1 larvae from each cross were tested on a diet with the diagnostic concentration of Cry1Ac (10 μg/mL) using the bioassay described above. We estimated the dominance parameter *h* in terms of survival at the diagnostic concentration adjusted for control mortality, which varies from 0 for completely recessive resistance to 1 for completely dominant resistance [11,35]. The dominance parameter *h* was calculated as the survival of F1 hybrid offspring–survival of *ss* divided by the survival of *rr*—survival of *ss*, with 0% for survival of *ss* according to the survival of the susceptible QJ-S strain. The survival of *rr* was based on survival for each resistant strain isolated from the F2 screening (R strain) and the survival of F1 hybrid offspring from crosses between each R strain and the susceptible strain (R strain × QJ-S). As an internal standard, the AQ47 (*r13r13*) strain was also crossed with itself and with the susceptible strain QJ-S.

### 4.6. Cloning and Sequencing the cDNA of Cadherin

We applied fourth instar larvae of the QJ43 and XZ27 strains that survived under the diagnostic concentration of Cry1Ac (10 μg/mL) to clone and sequence the full-length cDNA of *PgCad1*. We extracted total RNA using TRIzol (Invitrogen) and performed first-strand cDNA synthesis using M-MLV Reverse Transcriptase (Promega) according to the instructions. We carried out PCR amplification using the obtained cDNA as a PCR template and two pairs of specific primers based on conserved regions of the susceptible *s* allele of *PgCad1* (GenBank Accession AY198374.1) as described previously [24]. To determine the gDNA flanking sequences of mutation sites from the *r19* and *r20* allele, PCR amplification was performed using the primers r19-gF1/r19-gR1 and r19-gF2/r19-gR2 for the *r19* allele and r20-gF1/r20-gR1 and r20-gF2/r20-gR2 for the *r20* allele (nested PCR) (Table S1) and Phusion^TM^ Plus DNA Polymerase (Thermo). The PCR steps were as follows: 98 °C for 30 s, then 35 cycles including 98 °C for 10 s, 60 °C for 10 s and 72 °C for 4–5 min, and finally 72 °C for 5 min. Subsequently, the PCR products were purified, cloned, sequenced, and the gDNA and cDNA sequences of *r19* and *r20* were analyzed as reported previously [18].

### 4.7. Data Analysis

For bioassay data, we carried out probit regression by SPSS Statistics 22.0 to calculate LC_50_ values (concentration of Cry1Ac killing 50% of larvae) and their 95% confidence intervals (CI) and slopes of the concentration–mortality lines and their standard errors (SE) [36].

For the F2 screening, we calculated the frequency of resistance alleles as [valid single-paired lines with one resistance allele + 2 × (valid single-paired lines with two resistance alleles)]/(number of valid single-paired lines screened × 2 alleles per field male). We performed Fisher’s exact test to determine if the observed survival of F2 offspring was significantly different from the expected survival of F2 offspring.

## Figures and Tables

**Table 1:** Toxicity of Cry1Ac to pink bollworm larvae from the field-collected population (Anqing, Qianjiang and Xinzhou) and QJ-S laboratory susceptible strain.

Population	Slope (SE) ^a^	LC_50_ (95% CI) ^b^	RR ^c^	Survival at Diagnostic Concentration (10 μg/mL Cry1Ac) ^d^
QJ-S	3.78 (0.635)	0.077 (0.058–0.092)		0
Anqing	3.79 (0.645)	0.160 (0.125–0.189)	2.08	0
Qianjiang	4.23 (0.867)	0.180 (0.015–0.257)	2.34	0
Xinzhou	4.63 (0.931)	0.176 (0.028–0.245)	2.29	0

^a^ Slope of the concentration–mortality line with its standard error in parentheses. ^b^ Concentration killing 50% with 95% confidence intervals in parentheses, in μg Cry1Ac per ml diet. ^c^ Resistance ratio, the LC_50_ for the field-collected population divided by the LC_50_ for QJ-S. ^d^ For each population, the sample size is 72 at diagnostic concentration.

**Table 2:** Frequency of alleles conferring resistance to Cry1Ac in field populations of pink bollworm from China detected with the F2 screen.

Population	Total Single-Paired Lines Screened	Valid Single-Paired Lines Screened	Valid Single-Paired Lines Screened with Resistance	Resistance Alleles Detected ^a^	Resistance Allele Frequency ^b^ (95% CI) ^c^
Anqing	66	52	2	2	0.0192 (0.0033–0.0745)
Qianjiang	71	54	3	3	0.0278 (0.0072–0.085)
Xinzhou	56	39	2	2	0.0256 (0.0044–0.098)
Total	193	145	7	7	0.0241 (0.0106–0.0512)

^a^ See Table 3 for details. ^b^ Resistance alleles detected in valid single-paired lines divided by the total alleles screened in valid single-paired lines = [valid single-paired lines with one resistance allele + 2 × (valid single-paired lines with two resistance alleles)] divided by (number of valid single-paired lines screed × 2 alleles per field male). ^c^ The 95% Confidence interval.

**Table 3:** Observed and expected survival in F2 offspring of single-paired lines in which resistance to Cry1Ac was detected with the F2 screen.

		Survival at Diagnostic Concentration (10 μg/mL Cry1Ac)					
Single-Paired Line	n	Observed	Expected No.	Observed (%)	Observed vs. Expected (*p*)	Number of Resistance Alleles	Dominance and Recessiveness
AQ15	144	12	9	8.3	0.39	1	recessive
AQ39	96	3	6	3.1	0.25	1	recessive
QJ43	96	4	6	4.2	0.37	1	recessive
QJ58	120	9	7.5	7.5	0.40	1	recessive
QJ68	96	3	6	3.1	0.25	1	recessive
XZ27	144	8	9	5.6	0.62	1	recessive
XZ53	96	7	6	7.3	0.50	1	recessive

**Table 4:** Identification of resistance allele types of field-male parent in F2 survived single-paired lines.

Individuals ^a^	The Type of Resistance Allele ^b^					
	CK (Control)	*r1*	*r13*	*r14*	*r15*	*r16*
AQ15	+	−	+	−	−	−
AQ39	+	+	−	−	−	−
QJ43	+	−	−	−	−	−
QJ58	+	−	−	−	+	−
QJ68	+	+	−	−	−	−
XZ27	+	−	−	−	−	−
XZ53	+	−	+	−	−	−
AZP-R (*r1r1*)	+	+	−	−	−	−
AQ47 (*r13r13*)	+	−	+	−	−	−
AQ189 (*r14r14*)	+	−	−	+	−	−
JL46 (*r15r15*)	+	−	−	−	+	−
AQ65 (*r16r16*)	+	−	−	−	−	+

^a^ The AZP-R strain originated from Arizona with the cadherin genotype *r1r1*, while the AQ47, AQ189, JL46 and AQ65 strains were selected from the Yangtze River Valley of China with cadherin genotypes *r13r13*, *r14r14*, *r15r15* and *r16r16*, respectively [18,19,20,24,26]. ^b^ The plus sign “+” indicated positive, and the minus sign “−” indicated negative.

**Table 5:** Six transcript isoforms of eight disrupted cadherin alleles from the QJ43 and XZ27 strain.

Strain	Allele	Iso-Form	Deletion Size (bp)	Insertion Size (bp)	Cadherin Region	Premature Stop Codon (s)
QJ43	*r19*	*r19A*	1158	-	CR9-MPR	No
QJ43	*r19*	*r19B*	1157	-	CR11-CYT	Yes
QJ43	*r19*	*r19C*	1446	-	CR9-MPR	No
QJ43	*r19*	*r19D*	1956	-	CR9-CYT	No
XZ27	*r20*	*r20A*	881	94	CR4-CYT	Yes
XZ27	*r20*	*r20B*	1585	94	CR1-5	No

## Data Availability

The data that support the findings of this study are available from the corresponding author upon reasonable request.

## Supplementary Materials

The following are available online at https://www.mdpi.com/article/10.3390/toxins14010023/s1, Figure S1: Alignment of gDNA sequences of *r19* and *s* alleles; Figure S2: Alignment of gDNA sequences of *r20* and *s* alleles. Table S1: Primers used for cloning gDNA of *PgCad1*.
