# Supplementary material for: Mutation in the Cadherin Gene Is a Key Factor for Pink Bollworm Resistance to Bt Cotton in China
Source: Toxins (Basel). 2022 Jan 1;14(1):23. doi: 10.3390/toxins14010023 (PMC8777804; doi:10.3390/toxins14010023)
Supplement: Supplementary file 1 [file toxins-14-00023-s001.zip › toxins-1511477-supplementary.pdf]

toxins

MDPI

Supplementary Mutation in the Cadherin Gene Is a Key Factor for Pink Bollworm Resistance to Bt Cotton in China

Ling Wang, Dong Xu, Yunxin Huang, Huazhong Zhou, Weiguo Liu, Shengbo Cong, Jintao Wang, Wenjing Li and Peng Wan

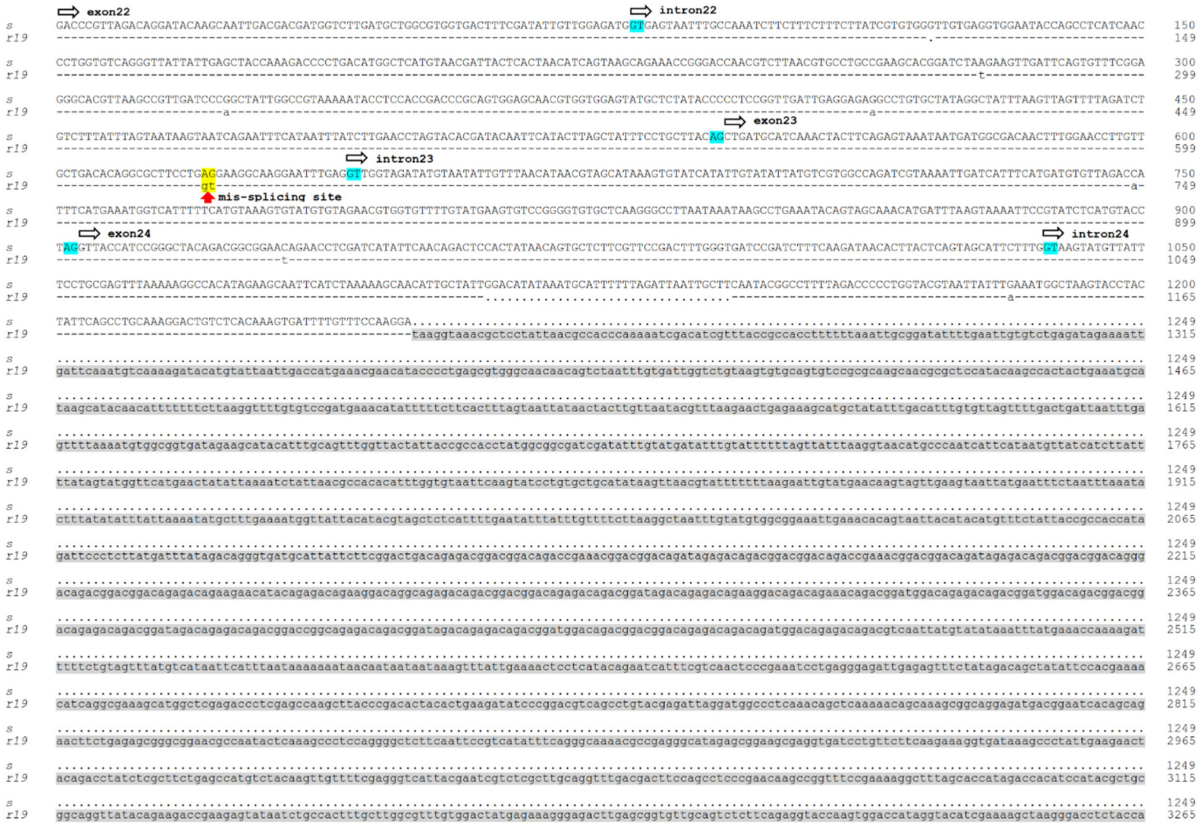

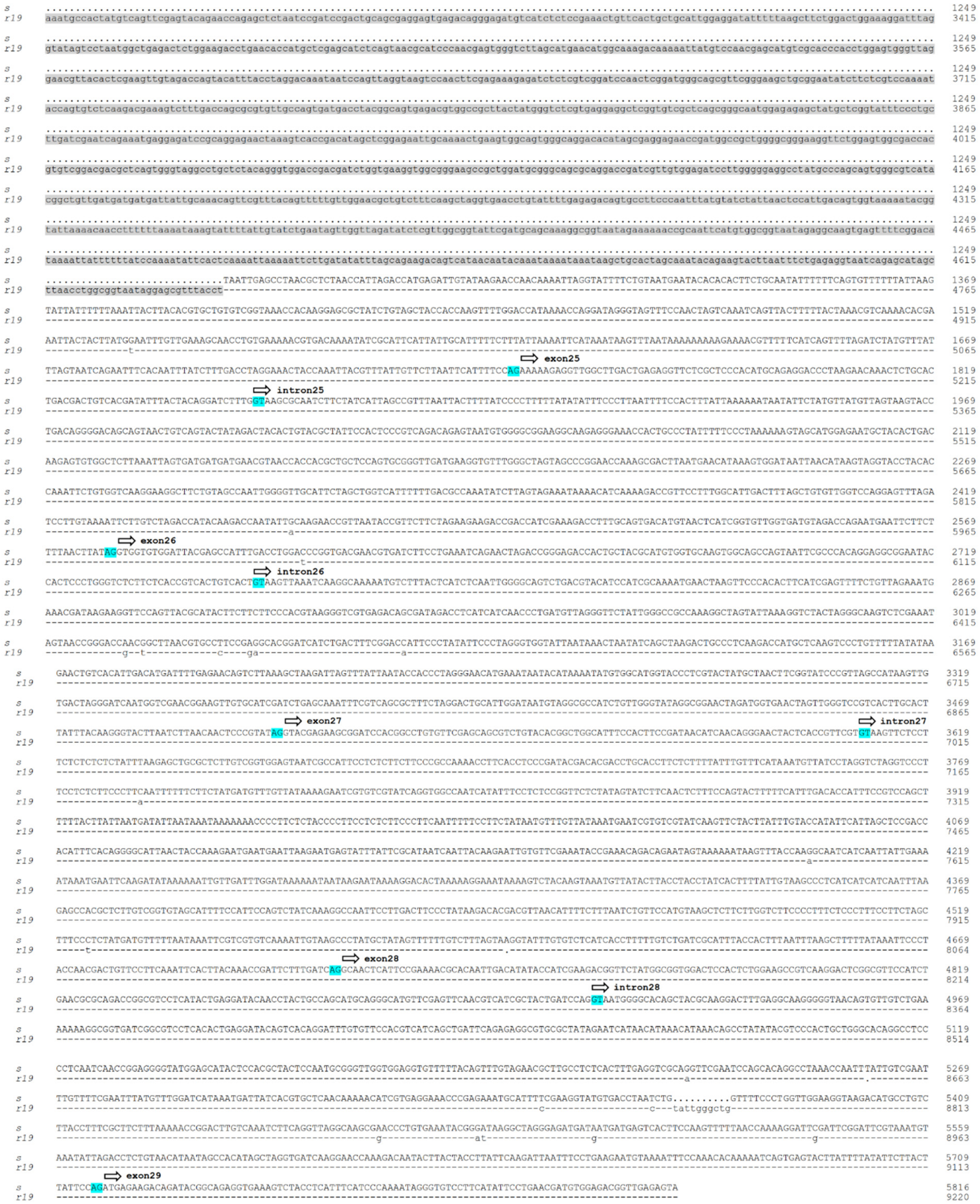

Figure 1. Alignment of gDNA sequences of *r19* and *s* alleles. The short dashes indicate a consistent sequence between *r19* and the *s* allele. The blue background GT/AG indicates a splicing site, and the right black arrow indicates the starting position of exons or introns. The yellow background AG/gt indicate the mutation site in the exon. The upward red arrow indicates the mis-splicing site in *r19*. The grey background indicates the 3431-bp inserted sequence in *r19*.

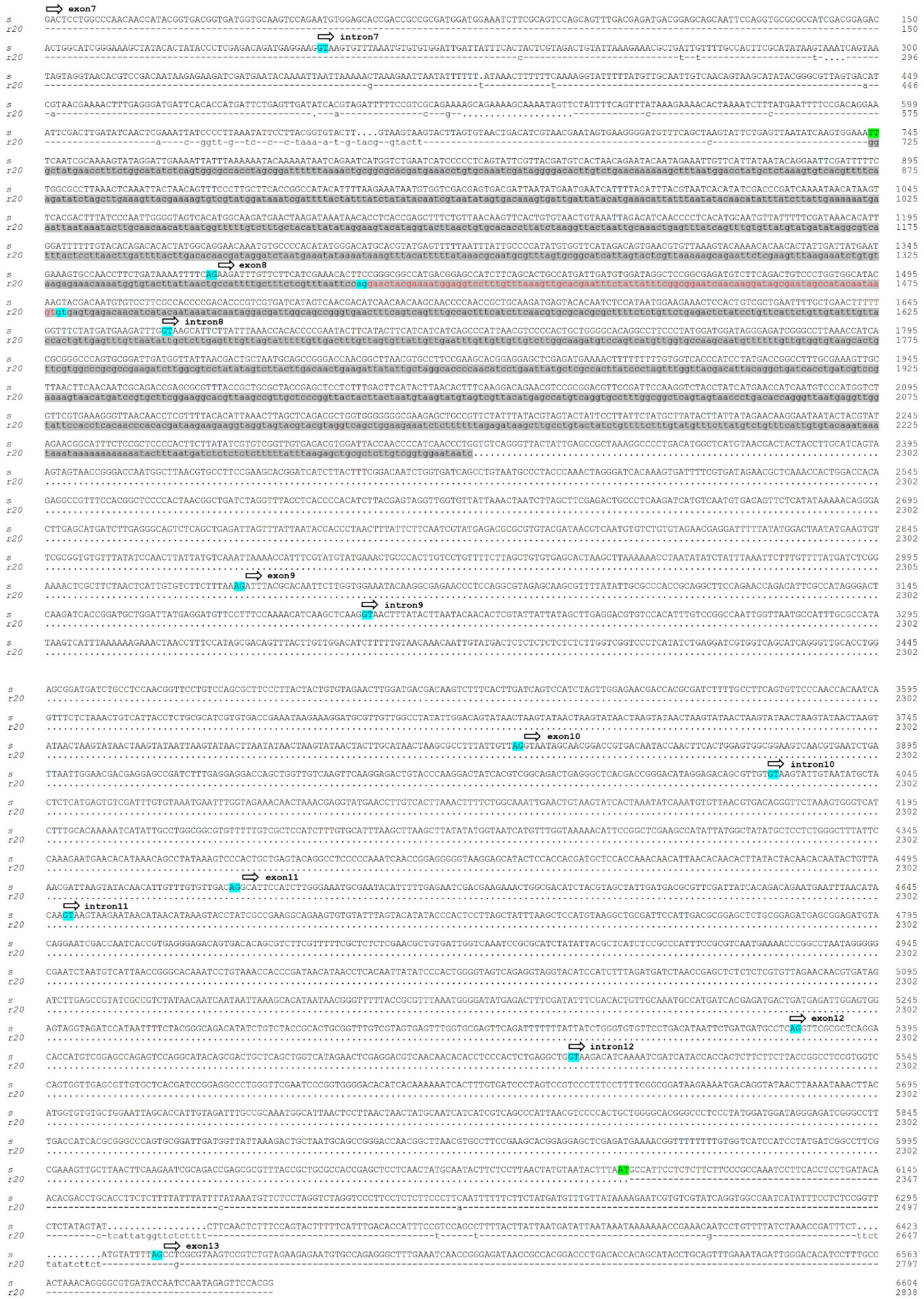

Figure 2. Alignment of gDNA sequences of *r20* and *s* alleles. The short dashes indicate a consistent sequence between *r20* and the *s* allele. The blue background GT/AG indicates a splicing site. The right black arrows indicate the starting position of the exons or introns in the *s* allele. The green background TT/AT indicates the starting point and endpoint of the 5357-bp deletion fragment in

r20. The grey background indicates the 1579-bp insertion fragment in *r20*. The red sequence indicates the 94-bp inserted sequence in the cDNA of *r20*.

Table 1. Primers used for cloning gDNA of *PgCad1*.

Name[a]	Primer sequence (5´-3´)
r19-gF1	GACCCGTTAGACAGGATACAAGCA
r19-gR1	CAGTGACGGTGAGAAGAGACCCAGG
r19-gF2	GTGGTGTGGATTACGAGCCATTTG
r19-gR2	TACTCTCAACCGTCTCCACATCGT
r20-gF1	GACTCCTGGCCCAACAACCATACG
r20-gR1	CCGTGGAACTCTATTGGATTGGTA
r20-gF2	CGCAGTCCAGCAGTTTGACGAGATG
r20-gR2	TCAGGGTCCGTGGCGGTTATCTC

[a]F indicates forward and R reverse.
